# The Effect of Oxytocin on Third-Party Altruistic Decisions in Unfair Situations: An fMRI Study

**DOI:** 10.1038/srep20236

**Published:** 2016-02-02

**Authors:** Yang Hu, Dirk Scheele, Benjamin Becker, Georg Voos, Bastian David, René Hurlemann, Bernd Weber

**Affiliations:** 1 Center for Economics and Neuroscience, University of Bonn, 53127, Germany; 2 Department of Psychiatry, University Hospital Bonn, 53105, Germany; 3 Department of Medical Psychology, University Hospital Bonn, 53105, Germany; 4 Key Laboratory for NeuroInformation, School of Life Science and Technology, Center for Information in Medicine, University of Electronic Science and Technology of China, 610054, P.R. China; 5 Department of Epileptology, University Hospital Bonn, 53127, Germany

## Abstract

Humans display an intriguing propensity to help the victim of social norm violations or punish the violators which require theory-of-mind (ToM)/mentalizing abilities. The hypothalamic peptide oxytocin (OXT) has been implicated in modulating various pro-social behaviors/perception including trust, cooperation, and empathy. However, it is still elusive whether OXT also influences neural responses during third-party altruistic decisions, especially in ToM-related brain regions such as the temporo-parietal junction (TPJ). To address this question, we conducted a pharmacological functional magnetic resonance imaging experiment with healthy male participants in a randomized, double-blind, cross-over design. After the intranasal administration synthetic OXT (OXT^IN^) or placebo (PLC), participants could transfer money from their own endowment to either punish a norm violator or help the victim. In some trials, participants observed the decisions made by a computer. Behaviorally, participants under OXT^IN^ showed a trend to accelerate altruistic decisions. At the neural level, we observed a strong three-way interaction between drug treatment (OXT/PLC), agency (self/computer), and decision (help/punish), such that OXT^IN^ selectively enhanced activity in the left TPJ during observations of others being helped by the computer. Collectively, our findings indicate that OXT enhances prosocial-relevant perception by increasing ToM-related neural activations.

Altruistic behavior – generally defined as acts which benefit another individual and confer costs for the actor – can be observed in various species^1^,^2^. Different from other animal species, however, human altruism also extends to unknown and genetically unrelated individuals^2^,^3^,^4^. For instance, humans across different cultures tend to help strangers in need even if they do not interact with them and will never personally meet them^5^.

One intriguing example of human altruism is third-party altruistic behavior, usually taking place in more complex situations in which three parties are involved. Specifically, third-party altruistic behavior in response to social norm violations (e.g. unfairness) can be one of two types. The third party (i.e. the observer) can enforce the social norm and help to restore social equity by either punishing the first party (i.e. the norm violator)^6^,^7^ or helping the second party (i.e. the victim of the norm violation)^8^,^9^. Interestingly, altruistic choices by the third party are also dependent upon individual trait differences in empathic concern, a trait involving other-oriented altruistic motivation^10^,^11^,^12^. Specifically, people with low empathic concern prefer punishment on the first-party violator, whereas those with high empathic concern more frequently choose to help the second-party victim^8^,^9^.

The hypothalamic peptide oxytocin (OXT) not only plays a key role in birth and lactation^13^,^14^ but also is a central modulator of social-emotional processing and even influences highly complex social behaviors, including pro-social behaviors and relevant domains, such as trust, cooperation, pair-bonding, and empathy^15^,^16^,^17^,^18^,^19^,^20^,^21^. While there is evidence that intranasal OXT (OXT^IN^) has pro-social effects such as increased trust^20^ (but also see^22^) and generosity^23^, recent findings emphasize that OXT’s pro-social effects strongly depend upon individual differences, such as personality traits^24^, and contextual factors, such as group membership^25^. With regard to altruistic behavior, a recent study reported that OXT^IN^ did not simply increase or decrease altruistic punishment in the ultimatum game, but rather reduced the sensitivity to contextual fairness^26^. Importantly, no study so far has investigated the effect of OXT on third-party altruistic behaviors.

A central prerequisite for altruistic decisions in complex third-party contexts is the capacity to understand others’ specific (affective) states, beliefs, and intentions, often referred to as theory-of-mind (ToM) or mentalizing^12^,^27^. On the neural level, ToM and mentalizing involve a broad neurociruitry centered around key nodes in the temporo-parietal junction (TPJ) and medial prefrontal cortex (MPFC)^28^,^29^,^30^. Previous behavioral studies have consistently documented that OXT^IN^ can enhance males’ performance in mentalizing and associated domains such as empathy^21^,^31^ and even in complex third-party contexts^32^, suggesting that modulatory effects of OXT^IN^ on ToM/mentalizing might underlie its proposed altruistic effects in third-party contexts. Besides, recent fMRI studies have also shown that both altruistic help and punishment choices elicit activations in a reward neurocircuitry involving the nucleus accumbens (NAcc). Specifically, NAcc responses were found when people donate their money to help unknown African orphans^33^ or when people punish an untrustworthy partner in a trust game^34^. In a third-party context, we also observed joint activation in NAcc and further striatal regions both when participants compensated the second-party victim or punished the first-party offender with their own endowment^9^. Since OXT^IN^ enhances NAcc responses in males viewing the faces of their female partners^19^, OXT may also modulate reward-related activations during third-party altruistic decision making.

In the present study, we have therefore endeavored to directly examine the effects of OXT^IN^ on third-party altruistic decisions and the underlying neural mechanisms. To this end, we combined OXT^IN^ with an fMRI paradigm on third-party altruism, which we had successfully implemented in a previous study^9^. During this paradigm, healthy male participants (i.e. the third party) were confronted with a series of unfair monetary offers made by different unknown proposers (i.e. the first party) to recipients (i.e. the second party). In the self-decision condition, participants were asked to make incentivized choices to either decrease the payoff of the proposer or to increase the payoff of the recipient with their own money. To further test the effect of OXT^IN^ on prosocial-relevant perception, we added another condition in which participants were only asked to observe computer-made decisions to punish the proposers or help the recipient. Based on the previous literature, we hypothesized that OXT^IN^ would increase third-party altruistic decisions and strengthen activations in a ToM-related network entailing the TPJ and MPFC^28^,^29^ during altruistic decision making. Based on findings from a recent behavioral study demonstrating that OXT selectively increased empathic responses to other’s pain from the other-perspective, but not from the self-perspective^35^, we further hypothesized that participants would show stronger TPJ and MPFC activity in OXT^IN^ condition during the observation of altruistic decisions made by the computer. Since previous studies also found that both third-party help as well as punishment decisions activate reward-relevant regions (e.g. NAcc)^9^,^33^, we hypothesized that OXT^IN^ would also increase activations in the NAcc during altruistic decision making. We also ran an exploratory analysis to investigate how individual differences in empathic concern influence the modulatory effect of OXT^IN^ on neural correlates of third-party altruistic decision making.

## Results

### Behavioral Results

The treatment had no influence on positive affect, negative affect, state anxiety, or the D2 attention performance (Table 1). Importantly, the subjects were not able to correctly identify whether they received OXT or PLC (correct estimates: OXT, n = 10; PLC, n = 14; χ^2^(1) = 0.376, p = 0.54). The drug treatment was counterbalanced across the two scanning sessions (session 1: OXT, n = 12; PLC, n = 10; χ^2^(1) = 0.364, p = 0.54). This suggests that confounding effects of OXT on mood, attention, or the subjective awareness of having received OXT can be excluded. For the post-scanning rating, no difference was detected between the two treatment conditions for 1) perceived unfairness of the offer, 2) perceived deservedness for punishing the first party, or 3) empathic concern for the second party (Table 1).

For behavioral measurements during the fMRI task (Table 2), two repeated-measure ANOVAs were performed with treatment (OXT/PLC) and decision type (help/punish) as within-subject variables and response time (RT; in ms) or transfer amount (in MU) as the dependent variable. Given the goal of the present study, we focused on the trials in which participants transferred at least 5 MU, namely the costly help or punishment choices. The analyses revealed a main effect of decision type on transfer amount (F(1, 21) = 6.295, p = 0.02, partial n^2^ = 0.231), showing that participants transferred more MUs when they decided to punish than to help. In addition, we detected a trend-to-significant effect (p < 0.1) of OXT suggesting that it slightly reduced the RT when people made prosocial decisions (F(1, 21) = 3.051, p = 0.095, partial n^2^ = 0.093). All other main and interaction effects failed to reach statistical significance (all ps > 0.16). As for the decision rate, paired samples t-tests were used to compare the percentage of the choices to help and to punish under OXT and PLC conditions, respectively. No significant differences were detected for either comparison (both ps > 0.7). To test whether individual differences in empathic concern can modulate the effect of OXT on choices, we calculated the differential decision rate (help vs. punish) between OXT and PLC conditions and correlated it with IRI_EC scores. No significant correlation was detected (r(20) = −0.083, p = 0.714).

### Imaging Results

**Figure 1:**
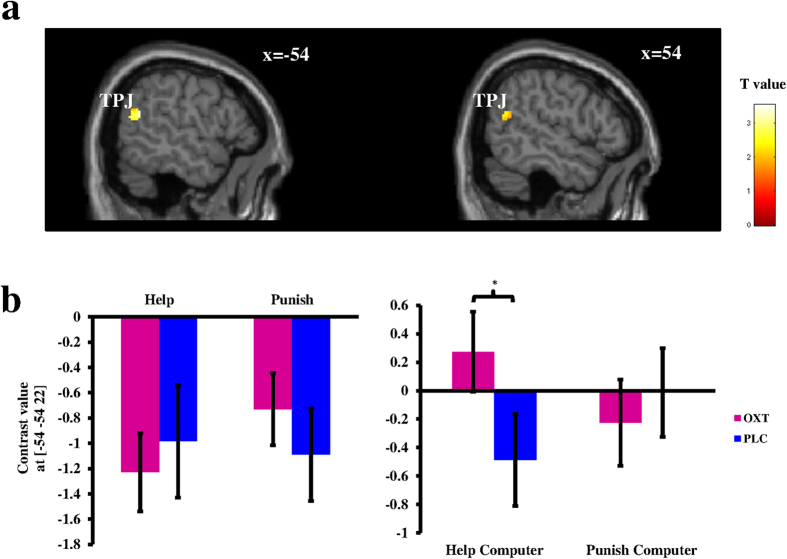
Three-way interaction between drug treatment (OXT/PLC), agency (self-decision/computer-decision), and decision (help/punish) in the TPJ. (**a**) Activitions for the contrast “[PLC_(help-help_computer)-(punish-punish_computer)] > [OXT_(help-help_computer)-(punish-punish_computer)]”. Display threshold was set at p < 0.05 uncorrected; (**b**) Plots of contrast values of the local peak voxel in the left TPJ. OXT = oxytocin, PLC = placebo; TPJ = temporo-parietal junction; ^*^p < 0.05; error bars: SEM.

To elucidate the specific OXT effect on the neural substrates of prosocial decisions made by the participant himself (i.e. the self-decision condition) and observing others being helped or punished (i.e. the computer-decision condition), we computed the contrast “PLC_[(help-help_computer)-(punish-punish_computer)] - OXT_[(help-help_computer)-(punish-punish_computer)]”. Within our hypotheses-driven, a priori ROIs, the interaction effect became significant in the left TPJ (peak MNI coordinates: −54/−54/22; t(63) = 3.54, p(FWE) = 0.005) and a similar trend was also observed in the right TPJ (peak MNI coordinates: 50/−58/20; t(63) = 2.35, p(FWE) = 0.079) as well as the MPFC (peak MNI coordinates: −2/56/20; t(63) = 2.25, p(FWE) = 0.095) (Fig. 1; for whole-brain results, see Supplementary Table S1). The reverse contrast yielded no activation in these regions. No significant activation was found in bilateral NAcc for either contrast. To further disentangle the three-way interaction effect in the left TPJ, we extracted the individual parameter estimates of the peak voxel from the separate conditions versus the implicit baseline (i.e. help, punish, help_computer, punish_computer separately for the OXT and PLC session) and ran two separate two-way repeated ANOVAs using parameter estimates for either self-decision or computer-decision trials, respectively. Post-hoc analyses revealed a strong interaction between treatment and decisions especially for computer-decision trials (F(1,21) = 10.536, p = 0.004, partial n^2^ = 0.334), driven by enhanced responses in left TPJ following OXT^IN^ while observing others being helped (t(21) = 2.348, p = 0.029; for punish_computer trials: t(21) = −0.782, p = 0.443; Fig. 1). In addition we detected a similar interaction effect in self-decision trials (F(1, 21) = 4.901, p = 0.038, partial n^2^ = 0.189). However, the post-hoc T-tests failed to reach significance for either altruistic choice (both ps > 0.16; for additional analyses, see Supplementary Table S2 and S3).

The whole-brain three-way ANOVA only revealed a strong main effect of agency. Specifically, motor-relevant regions, such as supplementary motor areas (BA 6/8), bilateral precentral gyrus extending to other parietal regions (BA 3/4/7), and decision-relevant regions, such as left middle frontal gyrus (BA 9/46), showed stronger activity during self- relative to computer-decisions. The reverse contrasts yielded significantly stronger activity for the computer- relative to the self-decisions in ToM-related regions, including the MPFC (BA 9/10), bilateral TPJ along with superior temporal gyrus (BA 21/22/39/40), and precuneus/posterior cingulate cortex (BA 7/31) (all regions mentioned here were significant at p < 0.05 whole-brain cluster-level family-wise error corrected with the uncorrected voxel-wise p < 0.001 and k = 100; see Supplementary Table S4 and Supplementary Fig. S1). No significant clusters in any other contrasts for either main effects or interactions were detected under the same threshold.

**Figure 2:**
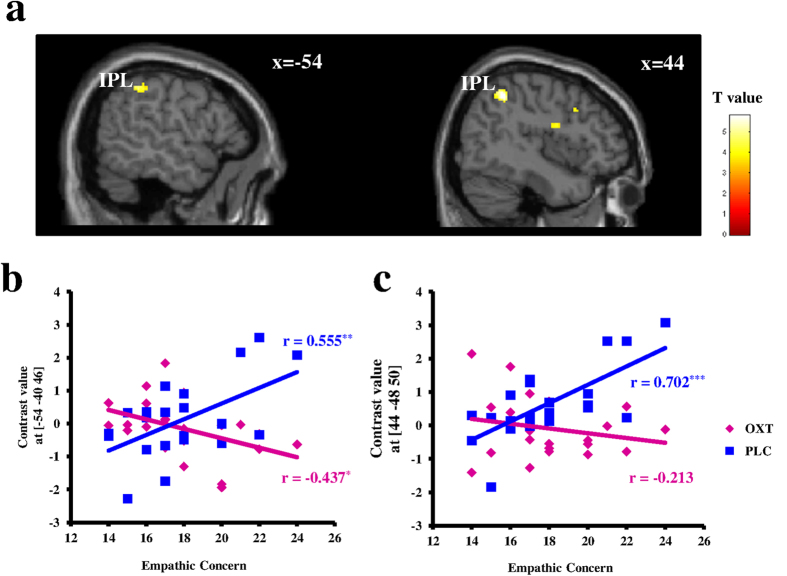
Modulatory influence of empathic concern on the effect of oxytocin on third-party prosocial decisions at the neural level (**a**) Neural responses for the contrast [PLC_(help-punish) - OXT_(help-punish)] positively correlated with empathic concern; the threshold was set at p < 0.001, k = 50; Plot of the positive correlation between empathic concern and contrast values in local peak voxel of the left (**b**) and right (**c**) lPL for the contrast help > punish under OXT and PLC. OXT = oxytocin, PLC = placebo; IPL = inferior parietal lobule; ^*^p < 0.05, **p < 0.01, ***p < 0.001.

A subsequent analysis examined whether trait empathy influences the effect of OXT (vs. PLC) on third-party pro-social decisions at the neural level. Interestingly, the exploratory whole-brain correlation analysis revealed a positive association between activities in bilateral inferior parietal lobules (IPL) of OXT^IN^ effects (i.e. the contrast [PLC_(help-punish)-OXT_(help-punish)]; for whole-brain results, see Supplementary Table S5) and higher trait empathy as assessed by IRI_EC scores (see Supplementary Table S6 and Fig. 2). To further explore this association, individual parameter estimates from the bilateral IPL peak voxels were extracted separately from the contrasts help vs. punish following OXT and PLC treatment (i.e. the contrast “help-punish” in both OXT and PLC conditions) and subjected to two separate correlations with IRI_EC scores. The results showed that the differential neural activities between help and punish decisions in the bilateral IPL positively correlated with IRI_EC scores following PLC only (left: r(20) = 0.555, p = 0.007; right: r(20) = 0.702, p < 0.001; Fig. 2) and that this relationship was blunted and even reversed following OXT (left: r(20) = −0.437, p = 0.042; right r(20) = −0.213, p = 0.342; Fig. 2).

## Discussion

In the present study, we aimed at elucidating the effects of OXT^IN^ on third-party altruistic decisions on the behavioral and neural level. On the behavioral level, OXT^IN^ had no effect on the frequency of help or punishment decisions, but it showed a trend to reduce the response time for making both help and punishment decisions compared with the PLC condition. On the neural level, OXT^IN^ selectively enhanced neural activations in the left TPJ in response to observations of others being helped by the computer. Furthermore, empathic concern positively correlated with neural responses to help decisions in the bilateral IPL compared to punish decisions only under PLC.

There is accumulating evidence that OXT can produce pro-social effects. For example, Kosfeld and colleagues (2005) first found that male participants treated with OXT^IN^ invested more to unknown trustees in a trust game^20^. Likewise, Zak *et al*. (2007) showed that OXT^IN^ increased human generosity in the ultimatum game^23^. Pro-social OXT effects are also, however, clearly dependent upon contextual factors^36^ and interindividual differences^24^. For instance, De Dreu and colleagues (2012) demonstrated in a series of experiments that OXT^IN^’s influence on cooperation substantially varies as a function of the group membership of others^25^. As such, the absence of an OXT effect on the frequency of altruistic decisions in the present study may be related to that fact that all players of the dictator game were strangers, in other words, “out-group”. No enhancement by OXT^IN^ on altruistic behaviors to out-group members was shown in previous studies^25^,^36^. Interestingly, we detected a trend-to significant effect of OXT^IN^ on reducing reaction time during altruistic decision making that might indicate that OXT enhances the salience of social information and promotes pro-social behavior as proposed by the social-salience hypothesis of OXT^37^,^38^. However, given that the effects did not reach statistical significance in the present study, this finding thus should be interpreted cautiously.

Numerous fMRI studies have documented the involvement of the TPJ and MPFC during ToM/mentalizing processes^28^,^29^,^39^. In line with these findings, our whole-brain analyses also revealed a stronger recruitment of the TPJ and MPFC during trials in which participants observed computer-decisions to help or punish compared with self-decision trials, indicating that a ToM/mentalization process was involved during observation. More importantly, we found that left TPJ was selectively activated when people observed the victims being helped by the computer only after they were treated with OXT^IN^. This finding was evident both in the excluded participants and in the entire sample (see Supplementary Table S2 and S3 for details). In fact, a study with brain-damaged patients found that the left TPJ is necessary for reasoning about the beliefs of others^40^. Several lines of research also suggest that the neuropeptide OXT plays a key role in mediating ToM/mentalizing. For instance, OXT^IN^ improved mind-reading, evidenced by a better performance in the Reading the Mind in the Eyes Test (RMET)^31^ (but see also^41^). Further supporting evidence for an OXT effect on ToM ability comes from two recent studies showing that participants under OXT^IN^ influence expressed more empathy when imagining others than when imagining oneself in pain^35^ and rated criminal offenses as more harmful for the victim^32^. Importantly, a recent fMRI study also detected positive associations between OXT plasma levels and neural activations in the left TPJ when observing the geometric shapes interacting in a goal-oriented fashion compared with shapes moving in a random fashion^42^. By using the RMET in the scanner, Gordon *et al*. (2013) demonstrated that OXT^IN^ also augments TPJ activations in a sample of children and adolescents with high-functioning autism spectrum disorder^43^. While the impact of genetic variations in the OXT system on the function and structure of the TPJ is still unclear^44^, our current finding together with previous studies point to OXT as a potent neuromodulator of ToM-associated activations in the TPJ, thereby facilitating pro-social perception. The fact that OXT^IN^ selectively enhanced left TPJ activation during computer-made help decisions is in accordance with the observations that OXT^IN^ caused women to treat computer partners more like humans^45^ and enhanced spontaneous anthropomorphism in women^46^. Our result suggests that such an effect of OXT^IN^ is also present in males and provides important insights on gender-specific and general effects of OXT.

Contrary to our initial hypothesis, we did not detect an effect of OXT^IN^ on neural responses in the NAcc, which is regarded as a core part of the human reward system^47^ and is involved in third-party altruistic decisions, as shown in our previous study^9^. However, this pattern of results is in line with our previous observations that OXT^IN^ does not unconditionally increase activations in brain reward regions^19^. In that study, we observed that OXT^IN^ specifically enhanced NAcc responses in men in response to the faces of their romantic partners, but not in response to similarly attractive unfamiliar women, suggesting that OXTs effects on the NAcc are mediated by the (social) context^19^. In fathers viewing the faces of their own children versus unknown children, OXT even reduced activations in reward-related brain areas^48^. Future studies are therefore warranted to elucidate whether OXT might alter NAcc activations during altruistic decisions relevant for proximate others (e.g. family members/friends vs. strangers, or unknown in-group/own-race members vs. out-group/other race members).

Moreover, OXT^IN^ altered the relationship between higher trait empathic concern and increased bilateral IPL activity during help compared with punish choices. The IPL constitutes a core component of the frontoparietal network, which plays a crucial role in top-down cognitive control and attention^49^,^50^. In our previous study^9^, we also observed a positive correlation between IPL activation in help compared to punish trials and empathic concern under baseline conditions, meaning that individuals with higher empathic concern may display a more pronounced differential IPL response between help and punish decisions. Under OXT^IN^’s influence, this link between empathic concern and IPL activation was blunted, suggesting that OXT might alter the attention driven by empathic concern towards the offender or victim in the third-party context and thus change the choices. Nevertheless, the role of IPL together with the modulation of OXT during this process remains unclear and needs to be clarified by future studies.

The present study has several limitations. First, to analyze a three-way interaction between treatment, agency, and decision, we could only use data from 22 out of 41 participants. Several participants did not show enough variability in their choices in either (or both) of the treatment sessions to define all necessary regressors in terms of their altruistic choices (i.e. help or punish). Although the effect of OXT^IN^ on left TPJ during computer-decision trials was similar across different samples, generalization of the current study is limited since findings are based on a sub-group of subjects who consistently showed altruistic choices in the current setting. Second, the present study recruited only healthy male participants. Since there are several other studies showing that OXT^IN^ can produce sexual-dimorphic effects^45^,^51^,^52^, our results cannot simply be extrapolated to females.

To sum up, the present fMRI with a modified third-party decision paradigm study provides the first evidence about the effect of OXT^IN^ on third-party altruistic choice and prosocial-related perception, based on a sample of healthy males. Given the requirement of fMRI data analyses, we focused on those participants who consistently showed altruistic choices (i.e. help/punish) in both treatment sessions. Specifically, OXT^IN^ shows a trend of accelerating pro-social decisions. Moreover, it selectively alters activations in the TPJ during the observation of computer-made help decisions, indicating that OXT^IN^ may facilitate altruism-related perception by enhancing the ToM system and inducing anthropomorphic tendencies in men. In addition, OXT^IN^ diminishes differences between individuals with low and high empathic concern in bilateral IPL activation during help (vs. punish) decisions. Taken together, our findings deepen the understanding towards the complex mechanism underlying OXT’s effect on human altruism and might also provide insights for the potential application of OXT in clinical practice, namely that OXT might help to normalize altered social behavior in patients with social and interaction difficulties (esp. autism).

## Methods

### Participants

Forty-one healthy German males (mean age = 25.10 ± 3.88 years) were recruited for the fMRI study. Screening of participants was conducted on a separate day before the fMRI session. The screening ensured that all participants were free of current or past psychiatric or neurological disorders, drug or alcohol abuse, or dependence and addiction to cigarettes, as assessed by medical history and a Mini-International Neuropsychiatric Interview^53^. Besides, participants also completed the empathic concern subscale of Interpersonal Reactivity Index (IRI) scale, which measures individual differences in empathic concern^54^. On the day of the fMRI assessment all participants were free of cold and were asked to maintain their regular bed/waking times, and abstained from caffeine and alcohol. The study was approved by the institutional review board (IRB) of the Medical Faculty of the University of Bonn and written consent was given by all participants according to the Declaration of Helsinki (BMJ 1991; 302; 1194). All experimental protocols and procedures were conducted in accordance with the IRB guidelines for experimental testing and were in compliance with the latest revision of the Declaration of Helsinki.

### Experimental Design

The research question was addressed by means of a pharmacological fMRI experiment in a double-blind, placebo-controlled, within-subject design. Specifically, each participant came twice for the experimental sessions and was randomly assigned to intranasal administration of either OXT (24 IU; Syntocinon-Spray; Sigma Tau; 3 puffs per nostril, each with ~4 IU OXT) or PLC (exactly the same only without the neuropeptide). The order of the drug administration was counterbalanced across participants. The average interval between the two experimental sessions was 17.4 ± 12.7 days.

### Scanning Procedure

Participants completed the State-Trait Anxiety Inventory (STAI) and Positive and Negative Affective Scale (PANAS) immediately before the drug administration and after the experimental task on both scanning days to control for potentially confounding effects of OXT on state anxiety and mood. A T1-weighted structural MRI (~6 min) was acquired before the drug administration on the first scanning day. Next, participants self-administered a single intranasal dose of 24 IU OXT or placebo. Participants received the instructions and practiced the task in a separate room before fMRI scanning.

The fMRI session started with an unrelated, non-aversive task (i.e. a 6-min resting state scanning) that will be reported in a separate publication and the third-party help/punishment (HelPun) paradigm started about 45 min after the drug administration. Before the fMRI paradigm, participants were firstly informed of another study, in which a group of participants were randomly assigned to the role of either the proposer (i.e. the first party) or the recipient (i.e. the second party) in a dictator game. Each proposer was endowed with 100 monetary units (MUs; 1MU = 20 cents) and could divide 100 MUs between him-/herself and the recipient, i.e. 50/50, 60/40, 70/30, 80/20, 90/10, 100/0. Unknown to participants, those offers were specifically created. Participants in the current study were informed that only the unfair offers (i.e. 60/40, 70/30, 80/20, 90/10, 100/0) would be forwarded to the third party (i.e. participants of the current fMRI study). Importantly, participants were then informed that they could influence the payoff of either the proposer or the recipient by investing their own endowment, meaning both options were costly for the participant. Specifically, they had to invest 1 MU to either subtract 3 MUs from the first party or add 3 MUs to the second party. They were also told that one trial would be randomly selected and the payoff in that trial would be used to pay the participant (i.e. 50 MUs minus the MU amount this participant transferred), the corresponding first party (i.e. original payoff minus triple the MU amount this participant transferred if the participant chose to *punish*), or the corresponding second party respectively (i.e. original payoff plus triple the MU amount this participant transferred if the participant chose to *help*).

**Figure 3:**
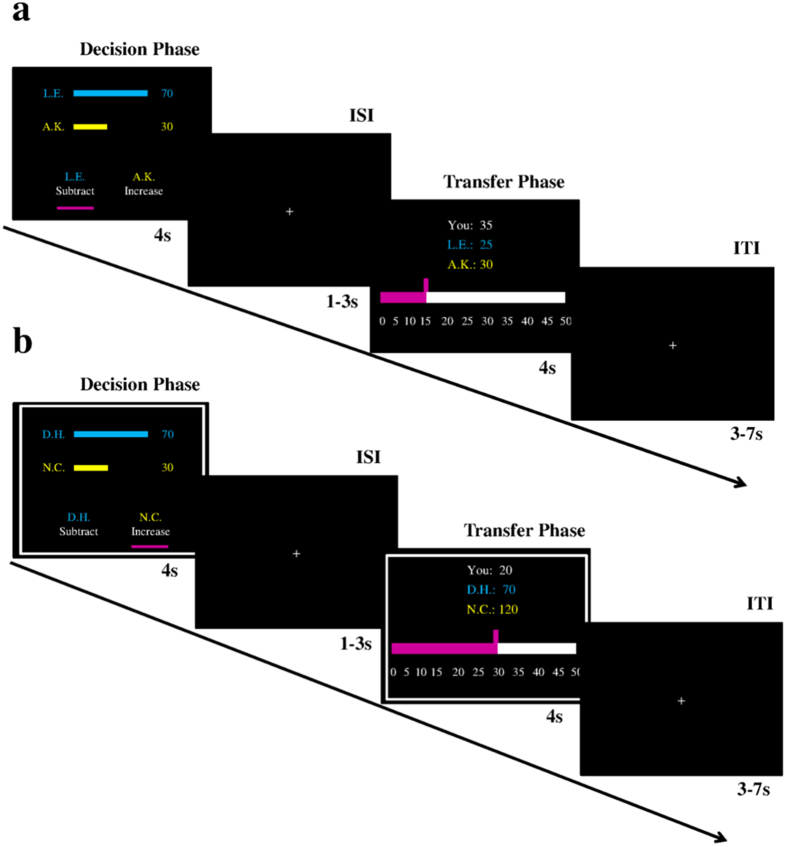
Illustration of trial procedures. (**a**) The self-decision condition and (**b**) the computer-decision condition. ISI = inter-stimulus interval; ITI = inter-trial interval.

The fMRI HelPun task consisted of 80 trials in the self-decision condition (16 trials per offer) and 40 trials in the computer-decision condition (8 trials per offer, half of them were in help/punish condition, respectively). In each trial, participants were endowed with 50 MUs (1MU = 20 cents). In the self-decision condition, participants first saw the unfair monetary allocation paired with the initials of both the first- and second-parties. On the same screen, they were asked whether they wanted to increase the recipient’s payoff or reduce the proposer’s payoff. Once they made a choice, a cue appeared under the corresponding option (i.e. the decision phase). The decision phase was presented for 4 s independent of their response time. The decision phase was followed by an inter-stimulus fixation cross (1–3 s). On the next screen, participants could decide how much they wanted to increase or decrease the payoffs of the other players (transfer phase; 4 s), followed by an inter-trial fixation cross (3–7 s). Participants could respond by pressing the response grips (Nordic NeuroLab, Bergen, Norway) with their left/right index fingers in both phases of the task. In the computer-decision condition, the procedure was identical except for a white frame, indicating that all decisions in those trials had been made by the computer (Fig. 3). No button presses were required in the computer-decision trials, however, participants still had an incentive to keep track of those trials since they were relevant for the payoffs of all three parties. The complete task lasted approx. 30 min.

Some methodological details were especially important to our paradigm. First, the words “help” and “punish” were not used in the instructions (“increase” and “subtract” were used instead) in order to avoid demand characteristics. Second, consistent with previous studies^9^,^55^, the cost ratio was set to 1:3, meaning that 1 MU could be used to either subtract 3 MU from the payoff of the first party or add 3 MU to the payoff of the second party. Third, participants could also decide to invest 0 MU during the transfer phase, meaning that the decision to increase or decrease other parties’ MU’s reflects participants’ voluntary decisions. Fourth, the position of two options (i.e. “increase” and “subtract”) in the decision phase were counterbalanced across trials. Fifth, the default position of the amount participants could invest in the transfer phase was randomly determined from 0 to 50 MU. Finally, the first party could not lose money; thus, the minimum payoff was 0. Importantly, all participants believed our cover story and none of them showed explicit doubt concerning the procedure and final payoff.

Stimuli were presented using Presentation 14 (Neurobehavioral Systems, Albany, CA) on a 32-inch MRI compatible TFT LCD monitor (NordicNeuroLab, Bergen, Norway) placed at the rear of the magnet bore.

### Post-scanning Task

After scanning, participants were asked to rate each offer they had seen on a 9-point Likert scale (0 = Not at all, 8 = very much) in terms of the following questions: 1) ‘How unfair is this offer?’, 2) ‘To what degree do you think the proposer deserves punishment?’, and 3) ‘How much empathy do you feel for the recipient?’. The order of the three questions was counterbalanced across participants. Participants then completed the D2 task to control for possible unspecific effects of OXT on attention. Finally, participants were asked to guess whether they had received OXT or PLC. After both sessions, participants received 60 € plus any extra money they earned during each session (around 25 € in total).

### Data collection

The imaging data was collected on a 3-Tesla Siemens Tim-Trio platform at Life & Brain Center, University Hospital Bonn. For functional images, 37 axial slices (FOV = 192 × 192 mm^2^, matrix = 96 × 96, in-plane resolution = 2 × 2 mm^2^, thickness = 3 mm) covering the whole brain were obtained using a T2*-weighted echo planar imaging (EPI) sequences with blood-oxygenation-level dependent (BOLD) contrast (TR = 2500 ms, TE = 30 ms, flip angle = 90°). A high-resolution structural image for each participant was acquired using 3D MPRage sequences for anatomical co-registration and normalization (TR = 1660 ms, TE = 2.75 ms, flip angle = 9°, matrix = 320 × 320, FOV = 256 × 256 mm^2^, slice thickness = 0.8 mm).

### Data quality assessment

Nineteen out of 41 participants were excluded for both behavioral and fMRI analyses due to the following reasons: 1 quitted the experiment session after feeling anxious in the scanner; 1 was given the same nasal spray twice; 1 showed extremely low scores in IRI empathic concern subscale (mean ± 3SD); 3 showed excessive head motion in both fMRI sessions (i.e. > 3 mm); 13 consistently showed only one or none of the altruistic decisions, resulting in a lack of target trials (fewer than 5 choices for each altruistic decision in one or both sessions) for any of the altruistic choices (i.e. 9 always chose to help; 1 always chose to punish; 3 always kept the money for themselves).

### Data analyses

Behavioral data were analyzed by using SPSS 22 (SPSS Inc.). Paired t-test as well as a repeated-measure analysis of variance (ANOVA) were used to test possible side effects and the effect of OXT on the behavioral indices. For ANOVAs, partial n^2^ was calculated as a measure of effect-size. Besides, Pearson correlation and chi-square test were also performed. All reported p values are two-tailed and p < 0.05 was considered significant.

fMRI data from the remaining 22 participants was analyzed with SPM8 (Wellcome Trust Department of Cognitive Neurology, London, UK). For each session of each participant, the first three volumes were discarded to allow for stabilization of BOLD signal. The following preprocessing steps were applied: EPI images were first realigned to the first volume to correct for head motions (<3 mm) and corrected for slice timing. Then, the anatomical image was co-registered to the mean EPI image and segmented, generating parameters for normalization to MNI space. Using these parameters, all EPI data were projected onto MNI space with a 2 × 2 × 2 mm^3^ resolution and smoothed using an 8-mm FWHM (full width half maximum) isotropic Gaussian kernel. High-pass temporal filtering with a cut-off of 128 s was performed to remove low-frequency drifts.

On the individual-level, a general linear model (GLM) focusing on the decision-phase convolved with the canonical hemodynamic response function (HRF) was applied. Separate regressors were included that modelled the onsets of the conditions “help”, “punish”, “help_computer”, “punish_computer”, and “other”. The “other” regressor included the following onsets: onsets of the transfer phase and onsets of no response as well as trials in which participants transferred 0 MU in the decision phase. The six estimated head movement parameters were included in the design matrix to account for the residual effects of head motion. For the group-level analyses, a random-effect flexible factorial model was designed with the factors of ‘treatment’ (OXT/PLC) and ‘difference between decision and computer trials’ (help-help_computer/punish-punish_computer) to test the three-way interaction between treatment (OXT/PLC), agency (self-decision/computer-decision), and choice (help/punish).

Given our prior hypotheses, we focused on two regions of interest (ROIs) as main components of the neural ToM network based on a recent meta-analysis^29^, namely the bilateral TPJ (5-mm sphere centered at these MNI coordinates: left: −53/−59/20; right: 56/−56/18) and MPFC (5-mm sphere centered at the MNI coordinate: −1/56/24). Given the key role of the NAcc for reward processing^47^, we also used ROIs of the bilateral NAcc, which were built with anatomical masks from the AAL template. The ROI masks were generated by the Wake Forest University Pickatlas (WFU) and the threshold for significance was set at p < 0.05 and familywise error (FWE) corrected for multiple comparisons based on the size of ROI. Parameter estimates (contrast values) of the peak voxel were extracted via MarsBar (http://marsbar.sourceforge.net). Main effects of the three factors and their two-way interaction effects (i.e. treatment x agency, treatment x choice, agency x choice) on the whole-brain were further explored using an additional unrestricted whole-brain three-way flexible ANOVA.

Furthermore, modulatory effects of trait empathy on the observed OXT^IN^ effects on third-party decisions were explored by means of an exploratory correlation analysis between the individual neural contrast of “[PLC_(help-punish)- OXT_(help-punish)]” and individual scores of the ‘empathic concern’ subscale of the IRI (IRI_EC). For all analyses above, we decided to report results from those 22 participants to allow the computation of the three-way ANOVA. For the whole-brain analysis, we adopted the threshold of p < 0.001 uncorrected at peak voxel level with an extent threshold of k = 50.

## Additional Information

**How to cite this article**: Hu, Y. *et al*. The Effect of Oxytocin on Third-Party Altruistic Decisions in Unfair Situations: An fMRI Study. *Sci. Rep.* **6**, 20236; doi: 10.1038/srep20236 (2016).

## Supplementary Material

Supplementary Information

## Figures and Tables

**Table 1:** State measurement of anxiety and mood and post-scanning ratings (N = 22).

	**OXT**	**PLC**	**Paired t-test**
	**Mean (S.D.)**	**Mean (S.D.)**	**t (p)**
***State Measurements***			
Positive affect pre	29.50 (5.91)	30.73 (5.49)	−1.144 (0.266)
Positive affect post	25.45 (6.44)	25.32 (7.45)	0.168 (0.868)
Negative affect pre	11.18 (1.01)	11.55 (1.79)	−1.250 (0.225)
Negative affect post	11.91 (2.54)	11.59 (2.13)	0.718 (0.481)
State anxiety pre	44.09 (1.44)	44.18 (2.34)	−0.153 (0.880)
State anxiety post	44.05 (2.77)	43.68 (1.89)	0.584 (0.565)
Attention	191.05 (78.00)	189.50 (86.96)	0.140 (0.890)
***Post-scanning Rating***			
Perceived unfairness of offer	4.73 (1.13)	4.95 (0.90)	−1.755 (0.094)
Deservedness for punishing the first-party	4.26 (1.39)	4.52 (1.00)	−1.161 (0.259)
Empathic concern for the second-party	4.43 (1.12)	4.62 (0.86)	−0.824 (0.419)

Note: OXT = oxytocin, PLC = placebo, pre = before treatment, post = after the scanning task; the PANAS was used for assessing positive/negative mood and STAI_state for state anxiety. Both mood and anxiety were measured before and after the treatment; the D2 test was used for assessing attention; all the post-scanning ratings ranged between 0 to 8.

**Table 2:** Behavioral measurements during the fMRI task (N = 22).

	Help		Punish	
	OXT	PLC	OXT	PLC
	Mean (S.D.)	Mean (S.D.)	Mean (S.D.)	Mean (S.D.)
Decision Rate (%)	52.67 (20.74)	53.64 (20.65)	38.30 (19.28)	37.16 (20.97)
Response Time (ms)	1630.76 (229.60)	1732.08 (399.76)	1680.41 (220.97)	1801.77 (427.63)
Transfer Amount (MU)	11.99 (5.92)	12.70 (5.86)	15.13 (5.57)	15.50 (5.82)

Note: OXT = oxytocin, PLC = placebo, MU = monetary unit.
