# Supplementary material for: The Effect of Oxytocin on Third-Party Altruistic Decisions in Unfair Situations: An fMRI Study
Source: Sci Rep. 2016 Feb 2;6:20236. doi: 10.1038/srep20236 (PMC4735743; doi:10.1038/srep20236)
Supplement: Supplementary Information [file srep20236-s1.pdf]

The Effect of Oxytocin on Third-Party Altruistic Decisions in Unfair Situations: An fMRI Study

Yang Hu[1,*], Dirk Scheele[2,3], Ben Becker[2,3,4], Georg Voos[1], Bastian David[1], René Hurlemann[2,3], Bernd Weber[1,5]

[1]Center for Economics and Neuroscience, University of Bonn, 53127, Germany

[2]Department of Psychiatry, University Hospital Bonn, 53127, Germany

[3]Department of Medical Psychology, University Hospital Bonn, 53127, Germany

[4]Key Laboratory for NeuroInformation, School of Life Science and Technology, Center for Information in Medicine, University of Electronic Science and Technology of China, 610054, P.R. China

[5]Department of Epileptology, University Hospital Bonn, 53127, Germany

[*]huyang@uni-bonn.de

Supplementary Information

Supplementary Table S1 Three-way interaction between drug treatment (OXT/PLC), agency (self-decision/computer-decision), and decision (help/punish) at the neural level (N = 22)

Brain Region	Hemisphere	Cluster Size	MNI Coordinates			BA	T-value
			x	y	z		
[PLC_(help-help_computer)-(punish-punish_computer)]-[OXT_(help-help_computer)-(punish-punish_computer)]							
SFG/MFG	R	234	32	16	54	6/8	4.07*
IFG/MFG	R	98	56	22	22	45/46	3.89
TPJ/SMG/STG	L	58	-52	-50	20	40	3.90
IPL	R	341	42	-46	46	40	4.18*
SMA/PaCL	B	337	-2	-12	70	6	4.67*
MTG	L	59	-44	-44	-8	37	3.85
PCG/PoCG	L	74	-54	-10	10	43	3.75
PCG/PoCG	R	141	52	-14	30	3/4	4.00
Thalamus	L	91	-18	-22	14		4.52
[OXT_(help-help_computer)-(punish-punish_computer)]-[PLC_(help-help_computer)-(punish-punish_computer)]							
-							

Note: Threshold is set to $p < 0.001$, k=50, uncorrected; * Significant at $p < 0.05$ family wise error corrected at the cluster level; OXT=oxytocin, PLC=placebo; L=left, R=right, B=bilateral, BA=Brodmann Area; IFG=Inferior Frontal Gyrus, IPL=Inferior Parietal Lobule, MFG=Middle Frontal Gyrus, MTG=Middle Temporal Gyrus, PCG=Precentral Gyrus, PoCG=Postcentral Gyrus, PaCL=Paracentral Lobule, SFG=Superior Frontal Gyrus, SMA=Supplementary Motor Area, SMG=Supramarginal Gyrus, TPJ=Temporo-parietal Junction; brain regions are labeled according to the automated anatomic labeling toolbox for SPM8.

Supplementary Table S2 Two-way interaction between drug treatment (OXT/PLC) and decision (help/punish) on computer-decision trials at the neural level (N = 34)

Brain Region	Hemisphere	Cluster Size	MNI Coordinates			BA	T-value
			x	y	z		
[PLC_(help_computer- punish_computer)]-[OXT_(help_computer –punish_computer)] - [OXT_(help_computer- punish_computer)]-[PLC_(help_computer -punish_computer)]							
PCG/IFG	L	179	-62	4	14	6/44	4.16
PoCG/Insula/STG	L	115	-48	-18	14	13/41/43	3.86
TPJ	L	255	-54	-52	26	40	4.38*
PaCL/SMA/PCG	B	399	2	-32	66	4/5/6	4.63*

Note: This additional analysis was conducted to test whether the modulatory effect of OXT^{IN} on computer-decision trials is biased by the exclusion of 13 participants who showed only one or none of the altruistic decisions (help/punish) in either OXT/PLC or both sessions. This analysis was performed in the entire sample (N = 34; 22 subjects in the main sample plus 12 additional subjects in the excluded sample; the fMRI data from one of the 13 excluded subjects were not available due to technical errors). By using the same ROI as in our main analyses (i.e. a 5mm-sphere centered around the coordinates of -53/-59/20), this analysis confirmed stronger activation of left TPJ (peak MNI coordinates: -54/-54/22; t(99)=3.77, p(FWE)=0.002) during computer-help decision trials in the OXT^{IN} condition (i.e. the contrast [OXT_(help_computer-punish_computer)-PLC_(help_computer-punish_computer)]). Post-hoc T-test further confirmed that OXT^{IN} selectively enhanced the activation in left TPJ during the help_computer trials (t(33) = 2.938, p = 0.006) rather than punish_computer trials (t(33) = -1.560, p = 0.128).

The whole-brain results are based on a threshold of p < 0.001, k=50, uncorrected; * Significant at p < 0.05 family wise error corrected at the cluster level; L=left, R=right, B=bilateral, BA=Brodmann Area; IFG=Inferior Frontal Gyrus, PCG=Precentral Gyrus, PoCG=Postcentral Gyrus, PaCL=Paracentral Lobule, SMA=Supplementary Motor Area, SMG=Supramarginal Gyrus, STG=Superior Temporal Gyrus; TPJ=Temporo-parietal Junction; brain regions are labeled according to the automated anatomic labeling toolbox for SPM8.

Supplementary Table S3 Two-way interaction between drug treatment (OXT/PLC) and decision (help/punish) on computer-decision trials at the neural level (N = 12)

Brain Region	Hemisphere	Cluster Size	MNI Coordinates x	y	z	BA	T-value
[PLC_(help_computer- punish_computer)]-[OXT_(help_computer -punish_computer)]							
-							
[OXT_(help_computer- punish_computer)]-[PLC_(help_computer -punish_computer)]							
IFG/Insula	L	99	-56	0	12	6/13/44	3.51
TPJ	L	372	-62	-48	32	40	3.71
IPL/SPL	R	56	48	-54	58	40	3.72
Insula/PoCG	L	233	-48	-18	16	13	3.74
Insula/PoCG	R	207	42	-16	22	13	3.92
PCG	L	145	-22	-12	60	6	3.42
PCG	R	185	32	-12	44	4/6	4.19
PoCG/PaCL/PCG	R	1009	48	-24	54	3/4/6	4.00*
TP/STG/PHG	L	332	-28	6	-24	28/38	3.95
STG	R	110	58	-16	8	22/41	3.35
MTG/ITG	R	83	64	-42	-10	21	3.40
FG	R	81	44	-34	-20	37	3.76
SMA/MCG	L	63	-10	-2	48	24	3.50
MCG/PaCL	L	137	-16	-24	42	24/31	3.41
PaCL	R	67	-16	-34	66	4/6	3.25

Note: This additional analysis was conducted to test whether the modulatory effect of OXT^{IN} on computer-decision trials is biased by the exclusion of 13 participants. This analysis was performed in the excluded sample (n = 12; see Supplementary Table S2). In the same ROI-based analysis, we also observed higher activation in left TPJ in the same contrast (see Supplementary Table S2; peak MNI coordinates: -48/-56/22; t(33) = 2.61, p(FWE)=0.05). Results of post-hoc T-test were also replicated in the 12 initially excluded subjects (help_computer trials: t(11) = 2.383, p = 0.036; punish_computer trials: t(11) = -1.236, p = 0.242). Together with the results in Supplementary Table S2, these findings were consistent with our results in the main analyses that OXT^{IN} selectively increased the activation of left TPJ during the observation of victims being helped by the computer, in the entire sample as well as in the sub-group of initially excluded subjects. To sum up, this suggests comparable effects of OXT^{IN} in the sub-groups and argues against specific effects in the sub-group selected by behavioral response in the main analyses.

The whole-brain results are based on a more lenient threshold given the smaller sample size (i.e. p < 0.005, k=50, uncorrected); *Significant at p < 0.05 family wise error corrected at the cluster level; OXT=oxytocin, PLC=placebo; L=left, R=right, B=bilateral, BA=Brodmann Area; FG=Fusiform Gyrus, IFG=Inferior Frontal Gyrus, IPL=Inferior Parietal Lobule, ITG=Inferior Temporal Gyrus, MCG=Mid-Cingulate Gyrus, MTG=Middle Temporal Gyrus, MOG=Middle Occipital Gyrus, PoCG=Postcentral Gyrus, PaCL=Paracentral Lobule, PHG=Parahippocampal Gyrus, SMA=Supplementary Motor Area, SPL=Superior Parietal Lobule, STG=Middle Temporal Gyrus, TP=Temporal Pole, TPJ=Temporo-parietal Junction; brain regions are labeled according to the automated anatomic labeling toolbox for SPM8.

Supplementary Table S4 Main effects of agency (self-decision/computer-decision) at the neural level (N = 22)

Brain Region	Hemisphere	Cluster Size	MNI Coordinates			BA	T-value
			x	y	z		
Self-decision - Computer-decision							
MFG/IFG	L	146	-38	48	8	10/46	4.05
MFG	L	410	-38	32	26	9/46	6.31*
IFG	R	216	60	10	24	9/45	4.50
Insula	R	240	34	20	8	13	5.61*
SMA/ACC/IPL/SPL/ Precuneus/PCG/PoCG/ Insula/ SFG/MFG	B	13095	-6	10	50	2/3/4/ 6/7/8/9/13/ 24/32/40	10.86*
MOG/IOG	L	377	-28	-90	-4	18/19	7.39*
MOG/IOG/MTG	R	556	32	-90	-4	18/19	7.08*
Thalamus/Brainstem	B	1052	-4	-26	-2		6.01*
Computer-decision - Self-decision							
SFG	B	361	4	46	48	8/9	4.74*
MPFC/SFG	B	588	-12	58	22	9/10	4.27*
IFG/MFG	R	678	50	42	2	45/46	6.33*
SFG/MFG	R	389	24	26	46	8	5.52*
TPJ/IPL/SMG/AG/ MTG/STG	L	1027	-48	-70	26	39/40	6.36*
TPJ/IPL/SMG/AG/ MTG/STG	R	4051	56	-52	18	21/22/ 39/40	7.97*
Precuneus/PCC/MCC	B	1036	-12	-52	32	7/31	4.90*
MTG/STG	L	1173	-56	-16	-8	21/22	5.63*
PoCG	L	122	-24	-40	60	3	3.88
Precuneus/PoCG	R	526	12	-50	62	3/5/7	4.81*
PHG/FG	L	319	-26	-50	-6	19	5.31*
PHG/FG	R	474	24	-44	-10	19	5.83*
Cuneus/LG/SOG/MOG	B	3692	-8	-94	12	7/17/ 18/19/31	8.92*
Hippocampus/ PHG/Amygdala	L	110	-24	-4	-14		4.20
Hippocampus/ PHG/Amygdala	R	124	20	-4	-14		4.69

Note: Threshold is set to $p < 0.001$, k=50, uncorrected; * Significant at $p < 0.05$ family wise error corrected at the cluster level; L=left, R=right, B=bilateral, BA=Brodmann Area; ACC=Anterior Cingulate Cortex, FG=Fusiform Gyrus, IFG=Inferior Frontal Gyrus, IPL=Inferior Parietal Lobule, IOG=Inferior Occipital Gyrus, LG=Ligual Gyrus, MCC=Mid-Cingulate Cortex, MFG=Middle Frontal Gyrus, MOG=Middle Occipital Gyrus, MPFC=Medial Prefrontal Cortex, MTG=Middle Temporal Gyrus, PCG=Precentral Gyrus, PoCG=Postcentral Gyrus, PHG= Parahippocampa Gyrus, SFG=Superior Frontal Gyrus, SMA=Supplementary Motor Area, SMG=Supramarginal Gyrus, SOG=Superior Occipital Gyrus, SPL=Superior Parietal Lobule, STG=Superior Temporal Gyrus; TPJ=Temporo-parietal Junction; brain regions are labeled according to the automated anatomic labeling toolbox for SPM8.

Supplementary Table S5 Two-way interaction between drug treatment (OXT/PLC) and decision (help/punish) on self-decision trials at the neural level (N = 22)

Brain Region	Hemisphere	Cluster Size	MNI Coordinates x	y	z	BA	T-value
[PLC_(help- punish)]-[OXT_(help-punish)]							
SMA/PaCL	B	128	-2	-12	70	6	4.44
Insula	R	102	46	2	4	13	4.27
Insula/PoCG	R	85	46	-18	18	13	3.67
STG/TPJ/MTG	L	153	-46	-50	10	39/41	4.51
PCG/STG	L	67	-54	-12	12	43	4.19
Precuneus/LG	R	85	16	-44	4	27	3.94
MOG	R	63	36	-70	-2	19/37	4.17
Thalamus	R	55	8	-18	2		3.92
[OXT_(help- punish)]-[PLC_(help-punish)]							
-							

Note: Threshold is set to $p < 0.001$, k=50, uncorrected; *Significant at $p < 0.05$ family wise error corrected at the cluster level; OXT=oxytocin, PLC=placebo; L=left, R=right, B=bilateral, BA=Brodmann Area; LG=Lingual Gyrus, MTG=Middle Temporal Gyrus, MOG=Middle Occipital Gyrus, PCG=Precentral Gyrus, PoCG=Postcentral Gyrus, PaCL=Paracentral Lobule, SMA=Supplementary Motor Area, STG=Superior Temporal Gyrus, TPJ=Temporo-parietal Junction; brain regions are labeled according to the automated anatomic labeling toolbox for SPM8.

Supplementary Table S6 Modulatory influence of empathic concern on the oxytocin effect on neural correlates of third-party prosocial decisions

Brain Region	Hemisphere	Cluster Size	MNI Coordinates			BA	T-value
			x	y	z		
[PLC_(help-punish) - OXT_(help-punish)] & Empathic Concern							
IPL	L	122	-54	-40	46	40	4.72
IPL	R	276	44	-48	50	7/40	5.77*
PCG/PoCG	L	92	-34	-26	52	3/4	4.37
[OXT_(help-punish) - PLC_(help-punish)] & Empathic Concern							
-							

Note: Threshold is set to $p < 0.001$, k=50, uncorrected; * Significant at $p < 0.05$ family wise error corrected at the cluster level; OXT=Oxytocin, PLC=Placebo; L=left, R=right, B=bilateral, BA=Brodmann Area; IPL=Inferior Parietal Lobule, PCG=Precentral Gyrus, PoCG=Postcentral Gyrus; brain regions are labeled according to the automated anatomic labeling toolbox for SPM8.

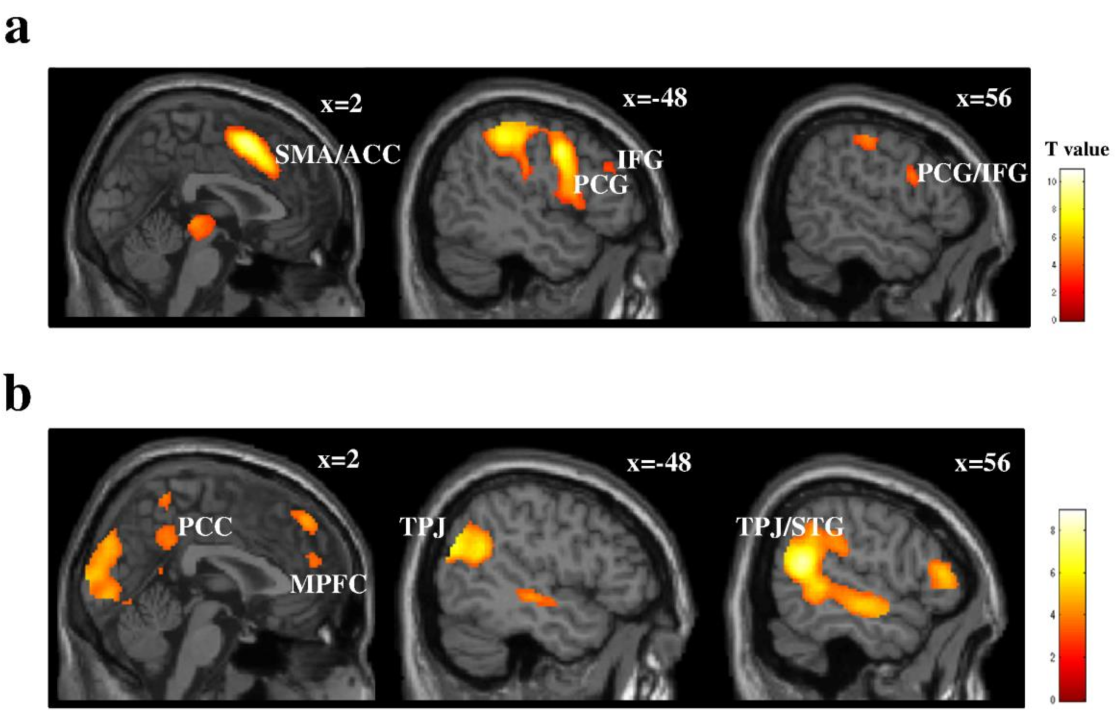

Supplementary Figure S1 Regions showing a main effect of agency (a) Regions showing enhanced responses in self-decision (vs. computer-decision) conditions; (b) Regions showing enhanced responses in computer-decision (vs. self-decision) conditions. ACC = Anterior Cingulate Cortex, IFG = Inferior Frontal Gyrus, IPL=Inferior Parietal Lobule, MPFC=Medial Prerontal Cortex, PCC = Posterior Cingulate Cortex, PCG = Precentral Gyrus, SMA=Supplementary Motor Area, STG = Superior Temporal Gyrus; TPJ=Temporo-parietal Junction; the threshold was set at $p < 0.001$, $k = 50$.
